# Expression of Carbonic Anhydrase I in Motor Neurons and Alterations in ALS

**DOI:** 10.3390/ijms17111820

**Published:** 2016-11-01

**Authors:** Xiaochen Liu, Deyi Lu, Robert Bowser, Jian Liu

**Affiliations:** 1 Department of Biological Sciences, Xi’an Jiaotong-Liverpool University, Suzhou 215123, China; xiao_chen_liu@yahoo.co.uk (X.L.); deyi.lu@xjtlu.edu.cn (D.L.); 2 Department of Neurobiology, Barrow Neurological Institute, Phoenix, AZ 85013, USA; Robert.Bowser@dignityhealth.org

**Keywords:** carbonic anhydrase 1 (CA1), amyotrophic lateral sclerosis (ALS), endoplasmic reticulum (ER), motor neuron, apoptosis

## Abstract

Carbonic anhydrase I (CA1) is the cytosolic isoform of mammalian α-CA family members which are responsible for maintaining pH homeostasis in the physiology and pathology of organisms. A subset of CA isoforms are known to be expressed and function in the central nervous system (CNS). CA1 has not been extensively characterized in the CNS. In this study, we demonstrate that CA1 is expressed in the motor neurons in human spinal cord. Unexpectedly, a subpopulation of CA1 appears to be associated with endoplasmic reticulum (ER) membranes. In addition, the membrane-associated CA1s are preferentially upregulated in amyotrophic lateral sclerosis (ALS) and exhibit altered distribution in motor neurons. Furthermore, long-term expression of CA1 in mammalian cells activates apoptosis. Our results suggest a previously unknown role for CA1 function in the CNS and its potential involvement in motor neuron degeneration in ALS.

## 1. Introduction

Carbonic anhydrases (CAs) are a large and ancient family of enzymes present in all organisms. CAs catalyze the naturally existing reversible reaction between the hydration (H_2_O) of carbon dioxide (CO_2_) and production of carbonic acid (H_2_CO_3_) in the form of bicarbonate ($H_{2}{\mathrm{CO}}_{3}^{-}$) and proton (H^+^) in living organisms. There are six genetic families of CAs: α-, β-, Υ-, δ-, ζ-, and η-CAs, with α-CA being the most recently evolved class and the one found mainly in mammals, and other families observed in the lower organisms [1].

Evolutionary analysis has revealed 17 isoforms in the α-CA family by sequence similarities and divergences [2]. These isoforms can be further grouped based on their diverse subcellular locations: cytosol (CA1,2,3,7,13), plasma membrane (CA4,9,12,14,15,17), mitochondrion (CA5a,5b) and extracellular space (CA6). The remaining members (CA8,10,11) are known as CARPs (Carbonic Anhydrase-Related Proteins) as they are catalytically inactive [2,3].

The intracellular pH of a cell is determined by the net contribution of acid-loading and acid-extruding mechanisms [4]. Ions that are involved in these mechanisms include Na^+^, H^+^, $H{\mathrm{CO}}_{3}^{-}$, and Cl^−^ that are transported in and out of cells by exchangers and transporters [5,6,7,8], whereas CO_2_ diffuses through cell membranes. Depending upon the concentrations of the molecules (CO_2_, H_2_O, $H_{2}{\mathrm{CO}}_{3}^{-}$, and H^+^), the physiological function of CAs is to dynamically regulate and maintain cellular pH homeostasis.

CAs are ubiquitously expressed with different isoforms present in specific cell types [6]. For example, both CA1 and CA2 are abundant proteins in blood. CA isoforms 2, 4, 12, 14 are expressed in the kidney where the transport of solutes and acid−base balance are highly regulated and maintained within the nephron [9]. CA3 is the predominant isoform expressed in skeletal muscles [10]. In the central nervous system (CNS), CA2 was initially known to be the dominant form [11,12,13,14], though recent studies have revealed that many additional isoforms including CA5,7,4,12,14,15 and CA8,10,11 are expressed in diverse cell types in the CNS [15,16,17,18,19,20,21].

In addition to the involvement in modulating, buffering, and maintaining of both intracellular and extracellular pH, CAs serve important functions in the neural transmission in the CNS. It has been demonstrated that during the synaptic transmission, the intracellular pH in the synaptic terminal undergoes a transient acidification followed by a prolonged alkalinization [22,23]. CA inhibitors caused an overall acidification of this process [22]. Depending on the expression of the specific isoforms, CAs in the CNS function in coupling the intracellular proton/bicarbonate cycle and lactate flux between glia and neurons as well as regulating the extracellular pH [24,25,26]. CAs have been shown to modulate both excitatory and inhibitory neuronal transmissions in hippocampus, cerebellum, and cortex [15,26,27,28,29]. Furthermore, CA4 and CA14 can be coupled to monocarboxylate transporters to modulate lactate flux between neurons and glial cells in energy consumption [24,26].

Defects in CAs result in the specific pathology and symptoms determined by the selective expression of the isoforms. *CA2*-deficiency in human causes osteopetrosis, renal tubular acidosis, and mental retardation [30]. Homozygous *CA9*-knockout mice have gastric hyperplasia and vacuolar degenerative changes in the brain with behavioral defects in locomotor activity and memory test [31,32]. On the other hand, *CA9* is highly expressed in several cancers [33]. Patients with a mutation in *CA12* exhibit hyponatremia and hyperchlorhydria [34]. Individuals with homozygous mutation in *CA5a* are reported to have lethargy, hyperlactatemia, and hyperammonemia during the neonatal period and early childhood [35]. CA6 is initially described as a gustatory protein and highly expressed in the salivary and mammary glands. Mice deficient in both copies of the *CA6* gene prefer bitter taste [36,37]. Polymorphism in the human *CA6* gene was also linked to bitter taste perception [37]. CARPs are predominantly expressed in neural tissues [38]. Patients with mutations in *CA8* show phenotype in cerebellar ataxia, mental retardation, and disequilibrium syndrome [19], while *CA8^−/−^* mice exhibit motor dysfunction and altered calcium dynamics in cerebellar granule cells [39]. Inactivation of *CA10* in zebrafish leads to abnormal embryonic development and altered movement pattern [40].

CA1 is a very early marker for erythroid differentiation and the second most abundant non-heme protein in erythrocytes [41]. Its expression is also detected in intestinal, vascular and corneal epithelia, synovium, and cardiac capillary endothelial cells [42,43,44,45,46]. CA1-immunoreactivity was observed in both Type I and II cells in the rat carotid body [47]. Only in one study, CA1 mRNA level in the mouse brain was reported to be extremely low compared with that of CA2 [48]. Therefore, whether CA1 is expressed in the CNS is unclear. To date, there have been a total of seven studies reporting CA1 being the key protein as a result of unbiased screenings between normal and pathological conditions [42,43,46,49,50,51,52]. The elevated CA1 level was found in the vitreous of diabetic retinopathy which contributes to retinal hemorrhage and erythrocyte lysis via prekallikrein activation [43,53]. The increased expression of CA1 found in the synovium of patients with ankylosing spondylitis may promote dysregulated calcification and bone resorption [42]. CA1 was found to be the major antigen in cecal bacterial Ag, which is associated with inflammatory bowel disease. The dendritic cell-mediated CA1-specific production of regulatory T cells can suppress the development of colitis induced by CD4^+^CD25^−^ T cells [52]. CA1, together with CA2, are increased in diabetic ischemic cardiomyopathy, and CA1 can affect apoptosis in vitro [46]. Similar to CA2, CA1 has been shown to be a potential novel biomarker for early stage of non-small cell lung cancer [54].

In the current study, we report CA1 expression in spinal cord motor neurons. In addition, a proportion of CA1s are associated with subcellular endoplasmic reticular (ER) structures. CA1 protein levels were preferentially increased in the spinal cord of patients with amyotrophic lateral sclerosis (ALS), while CA2 did not change in these same patients. Our in vitro cell culture data demonstrated that intracellularly expressed CA1 can induce apoptosis. Our study establishes CA1 expression in the human spinal cord and suggests that CA1 may have an important function in motor neuron degeneration in ALS.

## 2. Results

### 2.1. Carbonic Anhydrase I (CA1) Is Expressed in Human Spinal Cord Motor Neurons

Since CA1 has not been reported to be expressed in the CNS and we are interested in the potential function of CA1 in motor neurons as well as in motor neuron degeneration in the context of ALS-related pathology, we first examined whether CA1 is expressed in human spinal cord motor neurons. Because CA2 is known to be the most abundant CA isoform in the CNS and human CA1 (hCA1) shares 59.8% identity in the amino acid sequence with human CA2 (hCA2), we would like to be certain that the CA1 antibodies used in this study were CA1-specific and did not cross-react with CA2.

For this purpose, commercially available recombinant human CA1 (rhCA1) and CA2 (rhCA2) proteins were used in the Western blot analysis (Supplementary Materials, Figure S1). When an equal volume (4.5 μL) of the protein solutions was used, more than three-fold excess of CA2 protein (22.5 ng) over CA1 protein (6.42 ng) was observed on the gel (Supplementary Materials, Figure S1A, indicated by the intensity of SYPRO Ruby-stained band between Lane 1 and Lane 4). Four identical blots with an equal volume (9.5 μL) of CA1 and CA2 samples loaded for each lane were then probed with three different sources of the commercially available CA1 antibodies as well as a CA2 antibody. All CA1 antibodies recognized rhCA1 without any detectable cross-immunoreactivity to rhCA2 while the CA2 antibody recognized rhCA2 only (Supplementary Materials, Figure S1B). 

The specificity of one of the CA1 antibodies, HRP-GαCA1, was further examined using proteins from the human spinal cord (hSC). It so happened that the Broad Range Molecular Weight Standards used in our experiment contained bovine CA2 (bCA2). Bovine CA2 shares 58.0% and 80.4% amino acid sequence identity with hCA1 and hCA2, respectively. Though HRP-GαCA1 cross-reacted somewhat with bCA2, it recognized one hCA1 band in the spinal cord extracts (Supplementary Materials, Figure S1C, the left panel). In the meantime, the HRP-RbαCA2 antibody recognized one hCA2 band, but not hCA1, while also recognizing strongly the bCA2 because of the more shared identity (Supplementary Materials, Figure S1C, the right panel). Note that hCA2 is comprised of 260 amino acids and ran slightly faster than hCA1 (which contains 261 amino acids) on the SDS-PAGE. The slight difference in the sizes was readily visualized by drawing a horizontal dotted line across the center of the bands (Supplementary Materials, Figure S1D). Therefore, we conclude that the CA1 antibodies used in this study recognize hCA1 but not hCA2.

**Figure 1:**
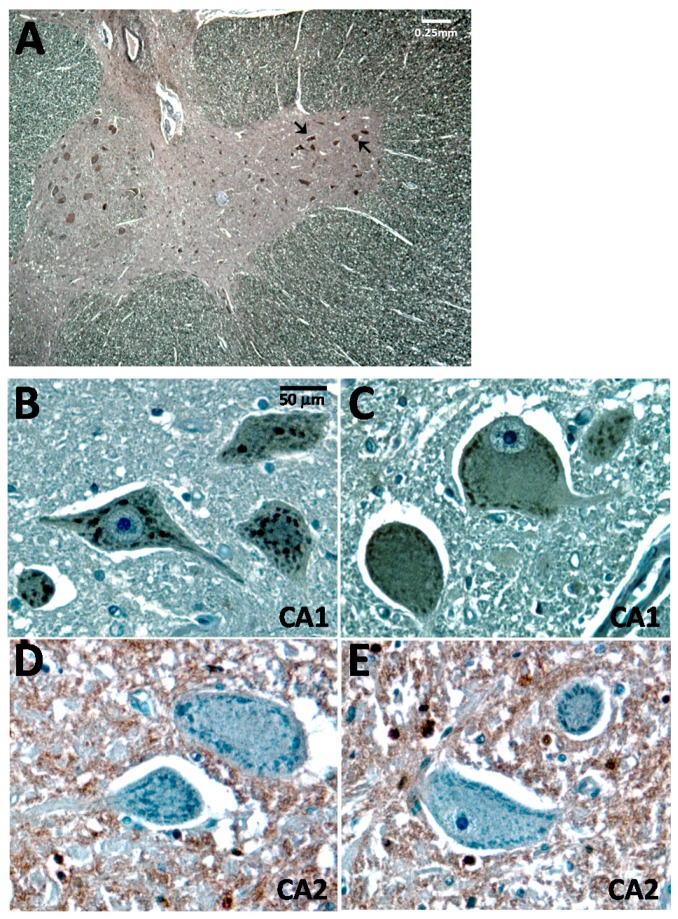
CA1 is expressed in human spinal cord motor neurons. Images of the normal human spinal cord immune-stained with CA1 or CA2 antibody using the DAB method (brown color) counter-stained with hematoxylin (blue color). The GαCA1 (1:500) and HRP-RbαCA2 (1:500) antibodies were used for this experiment. (**A**) A low magnification image of the ventral horn of spinal cord stained with the CA1 antibody. Two representative motor neurons are indicated by arrows. The white scale bar indicates 0.25 mm; (**B**,**C**) Higher magnification of spinal cord images stained with the CA1 antibody; (**D**,**E**) Higher magnification of spinal cord images stained with the CA2 antibody; The black scale bar indicates 50 μm for (**B**–**E**).

When control human spinal cord paraffin sections were immuno-stained with the CA1 antibody, the immunoreactivity was distinctively observed in large-sized motor neurons in the ventral horn (Figure 1A, also indicated by arrows). At a higher magnification, an unexpected punctuated pattern with a diffuse background staining was clearly evident (Figure 1B,C). When the HRP-RbαCA2 antibody was used to stain the adjacent spinal cord sections, the CA2-immunoreactivity was completely excluded from the large-sized motor neurons (Figure 1D,E). This observation is consistent with the notion that CA2 isoform is mainly expressed in oligodendrocytes and astrocytes [18], though the accurate demonstration of CA2 expression in the spinal cord was not further determined in our study.

### 2.2. A Subpopulation of Neuronal CA1 Appears to Be Associated with the Endoplasmic Reticulum Subcellular Structure

**Figure 2:**
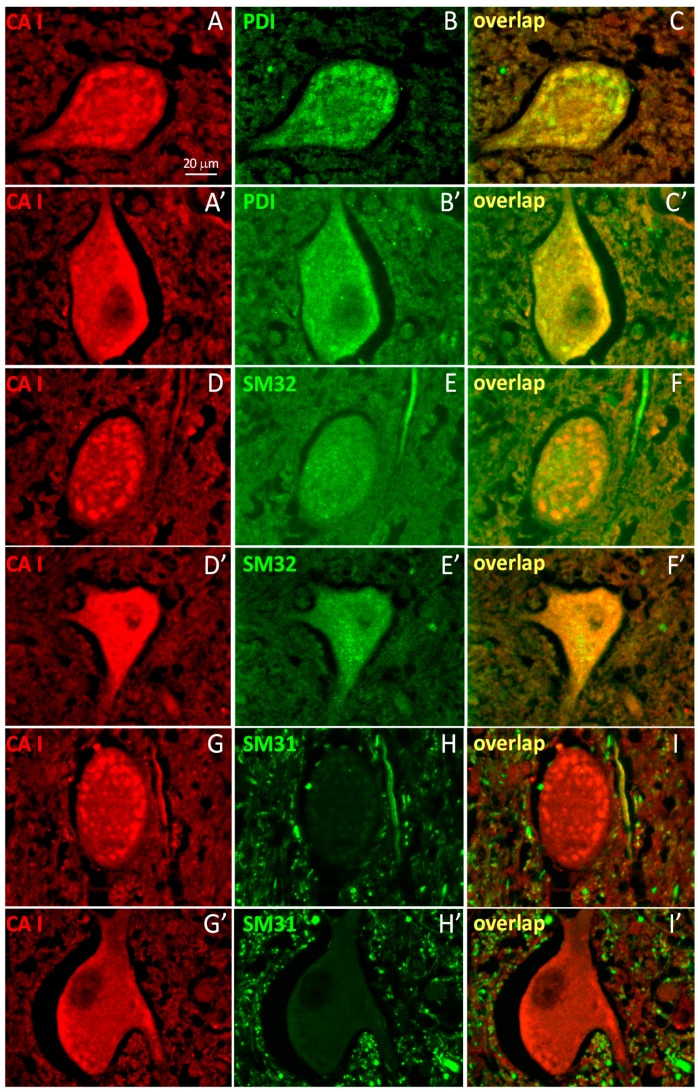
The punctate CA1-immunoreactivity co-localizes with the ER marker in control spinal cord motor neurons. Images are motor neurons double-labeled with fluorescent antibodies against CA1 (**red**, GαCA1, 1:100) and PDI (**green**) or neurofilaments (SM31, SM32, **green**) and the overlapped signals are shown in the far right panels. Images were from spinal cord sections of three control subjects (Supplementary Materials, Table S1, A–C) Two representative motor neurons of each double-labeling set are presented: CA1 and PDI (**A**–**C**,**A’**–**C’**); CA1 & SM31 (**D**–**F**, **D’**–**F’**); and CA1 and SM32 (**G**–**I**, **G’**–**I’**). The white scale bar indicates 20 μm.

**Figure 3:**
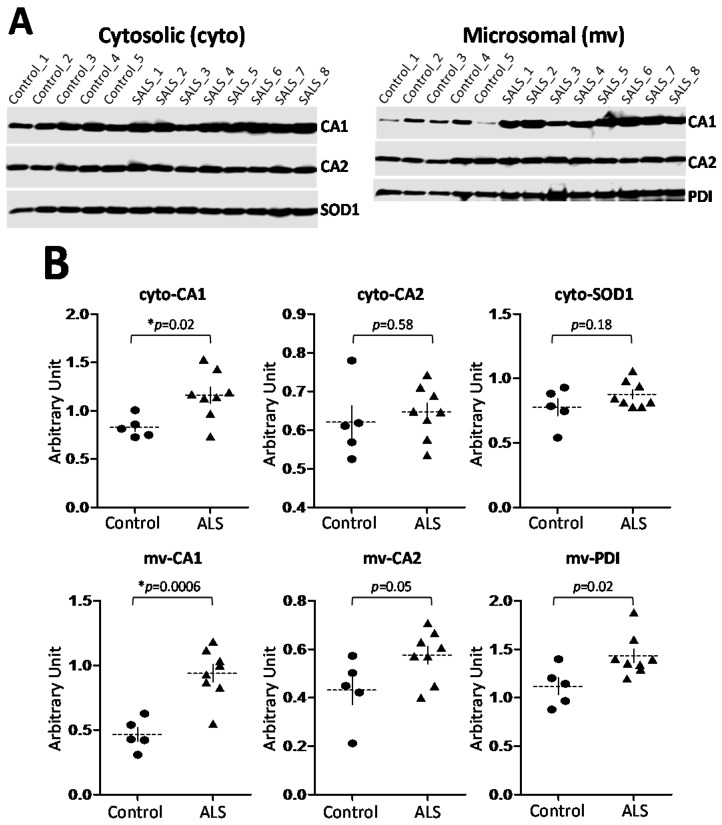
CA1 is differentially regulated from CA2 in ALS spinal cord. (**A**) Western blot analysis of the proteins from either the cytosolic (cyto) or microsomal (mv) fractionation extracted from the spinal cords of the control or ALS subjects probed with CA1 (HRP-GαCA1, 1:5000), CA2 (HRP-RbαCA2, 1:5000), SOD1, and PDI antibodies. An equal amount of proteins were used for each lane for either “cyto” or “mv” fraction; (**B**) Quantitative analyses of the differences in the intensities of immune-reactive signals for each protein between the control and ALS groups. All data points were indicated for each group. The dotted horizontal and solid vertical lines in each group represent “Mean ± SD” of the group value. *p* values are indicated for each graph, and * indicates *p* < 0.05.

To further characterize the subcellular structures represented by the punctate CA1-immunoreactivity, the staining patterns of molecular markers from subcellular organelles including the mitochondria, endoplasmic reticulum (ER), Golgi, endosomes and lysosomes were compared with that of CA1 (see some examples in Supplementary Figure S2). When it was obvious that the CA1 staining pattern most resembled that of an ER-marker PDI (protein disulfide isomerase), double-labeling for co-localization was further conducted for PDI and CA1 antibodies only. Indeed, PDI-labeling was found to co-localize with CA1-labeling (Figure 2A–C,A’–C’). To further demonstrate the relevance and the new feature of the CA1-immunoreacitivty, two neuronal markers SM32 (against cytosolic non-phosphorylated neurofilaments) and SM31 (against phosphorylated neurofilaments present predominantly in axons) were used to double-label spinal cord sections with the CA1 antibody. The diffusive pattern of CA1-immunoreactivity overlapped with that of SM32 (Figure 2D–F,D’–F’) but not SM31 (Figure 2G–I,G’–I’). The pattern of the CA1-immunoreactivity in the spinal cord demonstrated that CA1 is expressed in motor neuron soma while CA2 is present predominantly in non-neuronal cells. In addition, a portion of CA1 proteins in the spinal cord neurons appear to be associated with the ER structure. This is corroborated by the biochemical property of CA1 found in the 100,000× *g* membrane-fraction (Figure 3, microsomal or “mv”).

### 2.3. CA1 Is Preferentially Elevated in ALS Spinal Cord

To further determine the potential function of CA1 in motor neurons, we examined CA1 protein levels in the context of motor neuron degeneration in ALS spinal cord. Human spinal cord proteins were extracted as two fractions: cytosolic (cyto) and microsomal (mv) from both non-neurologic disease controls and sporadic ALS (SALS) patients (Supplementary Materials, Table S2 for information on the samples). The establishment and validation of this fractionation methodology was described in detail in a previous published study [55]. Briefly, this differential centrifugation-based fractionation method removes nuclei and mitochondria sequentially. The final step is the separation of the cytosolic (cyto) proteins from the membrane fraction (mv) by 100,000× *g* centrifugation. Membrane structures from subcellular organelles other than nuclei and mitochondria should remain in the “mv” fraction. Consequently, proteins including PDI are readily detected in the “mv” fraction (Figure 3A).

Initially, we determined the levels of cytosolic CA1s in larger sizes of control and ALS samples (Supplementary Materials, Figure S3). To ensure the quality of an equal amount of sample loaded for each lane, three parameters including the total amount of proteins in each lane visualized by SYPRO Ruby-staining and the intensities of the immunoreactive signals of both actin and another abundant cytosolic protein copper, zinc superoxide dismutase (SOD1) were used as the references for CA1 quantification (Supplementary Materials, Figure S3A,B). As expected, there is no significant difference between the control and ALS groups in each of the above three parameters, while CA1 level was significantly increased in ALS spinal cord (Supplementary Materials, Figure S3C).

Subsequently, the levels of both CA1 and CA2 proteins were determined for either “cyto” or “mv” fractions in smaller sizes of the samples due to the insufficient available spinal cord materials from some subjects for the “mv” analysis. In these experiments, SOD1 was used as the internal control for the “cyto” fraction, as its level is not expected to change in all samples as demonstrated (Supplementary Materials, Figure S3). In this reduced cohort, CA1 but not CA2 protein levels were increased in ALS patients (Figure 3); as expected, the level of cytosolic SOD1 protein did not differ between the two groups (Figure 3A, the left panel; Figure 3B, cyto-SOD1). CA2 levels were not significantly changed in either the “cyto” or “mv” fraction between the control and ALS samples (Figure 3B, cyto-CA2 and mv-CA2). Interestingly, the membrane-associated CA1 (mv-CA1) was increased more pronouncedly when compared to the cytosolic CA1 in ALS spinal cord (Figure 3B, cyto-CA1 vs. mv-CA1). The ER-resident protein PDI in the “mv” fraction was also examined and it was found to be increased in ALS spinal cord (Figure 3A, the right panel and Figure 3B, mv-PDI). Our data is consistent with the published data demonstrating that PDI was upregulated in ALS patients [56].

### 2.4. Altered Patterns of CA1 Expression in ALS Pathology

**Figure 4:**
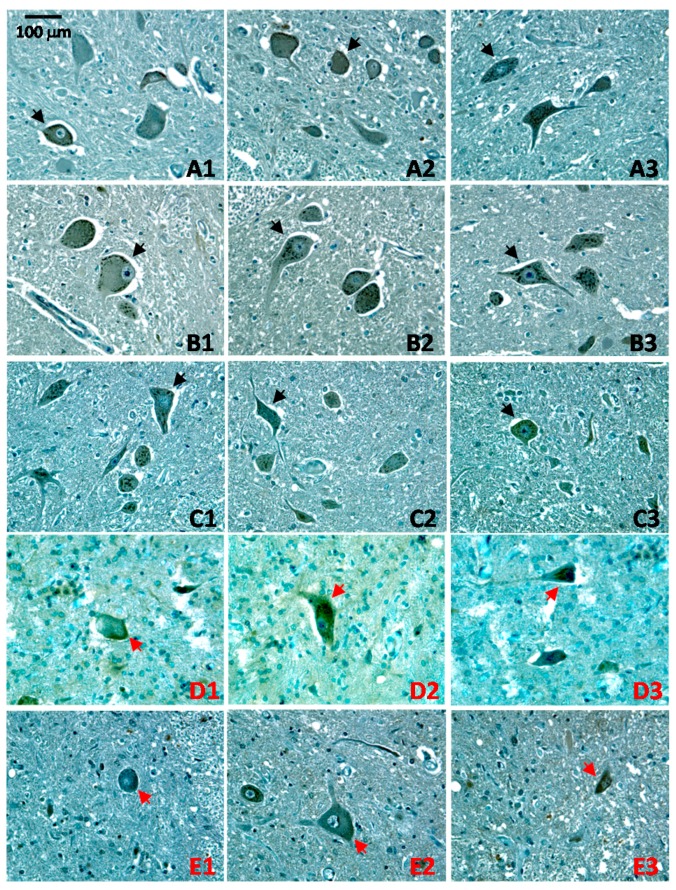
Altered CA1-immunoreactive patterns in ALS pathology. Spinal cord sections were immunohistochemically stained for CA1-immunoreactivity (brown, GαCA1, 1:500) and counter-stained with hematoxylin (**blue**). Three randomly selected images (indicated by 1–3) were shown for each sample. Samples labeled in black (**A**–**C**) are from control subjects; and those labeled in **red** (**D**–**E**) are from ALS patients. Neurons with the normal punctate CA1-immunoreactive distribution are indicated by black arrows and those with altered CA1-immunoreactive pattern were indicated by red arrows. The black scale bar indicates 100 μm.

To further characterize CA1 in ALS, we next examined the patterns of CA1 expression in ALS spinal cord motor neurons by immunohistochemistry (see Supplementary Materials, Table S1 for information on the samples). Compared to the spinal cords from control subjects (Figure 4A–C), a different CA1-staining pattern was observed in almost all the fewer remaining neurons in ALS spinal cords (Figure 4D,E).

The typical punctate staining pattern in the cytoplasm of motor neurons (Figure 4, indicated by black arrows) was lost in the remaining motor neurons in ALS spinal cord (Figure 4, indicated by red arrows). The altered CA1 appearances in ALS spinal cord can be the consequences of the overall degeneration and damage of motor neurons in ALS pathology.

### 2.5. Long-Term CA1 Expression Induces Apoptosis in HEK293 Cells

**Figure 5:**
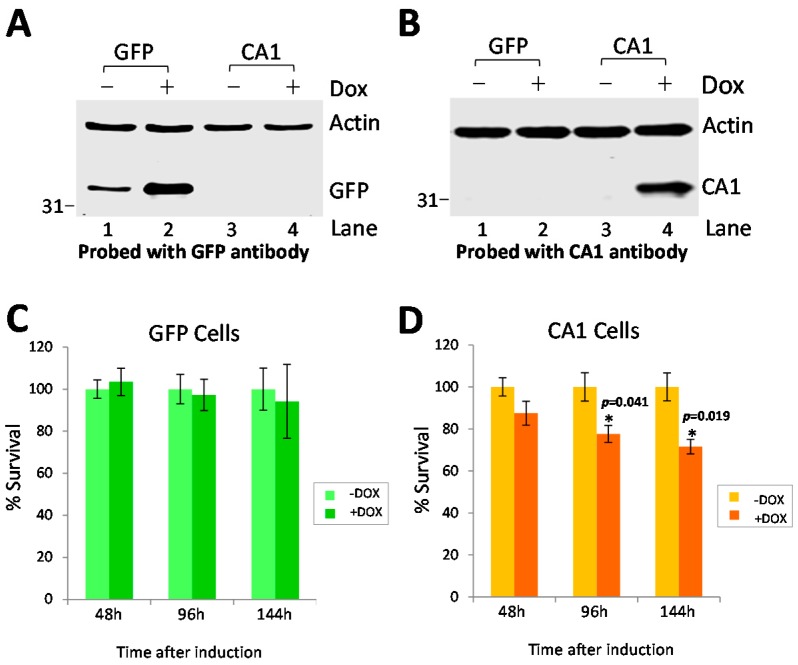
Long-term expression of CA1 decreases HEK293 cell survival. The expression of either GFP or CA1 was induced by 0.25 μg/mL doxycycline (DOX) in HEK293 cells at different times. The expression of GFP (**A**) and CA1 (**B**) in absence (−) and presence (+) of DOX was examined at 96 h by Western blot analysis; CA1 antibody (ab1088367, 1:5000) was used for (**B**); The rate of cell survival was measured by the WST8 assay at the indicated time (48, 96 and 144 h, respectively) in absence (−) and presence (+) of DOX for GFP (**C**) and CA1 (**D**). The data are the average of three independent experiments and expressed as “Mean ± SEM”. * indicates *p* value < 0.05. The *p* values (not indicated by *) for GFP at 48, 96, 144 h, and CA1 at 48 h are 0.68, 0.80, 0.20, and 0.15, respectively.

In order to assess whether the changes in CA1 can contribute to or compensate for ALS pathology, we overexpressed CA1 in HEK293 cells and examined its effects on cell survival and/or apoptosis. Overexpression of GFP in HEK293 cells was used as control. The expression of either GFP or CA1 in these cells was confirmed by Western blot analysis at 96 h post-induction (Figure 5A,B). While there is a leaky expression of GFP in the inducible stable GFP cell line, the presence of doxycycline (DOX) induced detectable expression of both GFP and CA1 (Figure 5A, Lane 2 and Figure 5B, Lane 4, respectively). Cell survival was measured by the WST8 assay after induction of CA1 expression at different times. While no significant toxicity resulted from induction of GFP expression in HEK293 cells at the indicated times (Figure 5C), expression of CA1 caused reduced survival at later times (Figure 5D, both 96 h and 144 h), but not at an earlier time (Figure 5D, 48 h).

**Figure 6:**
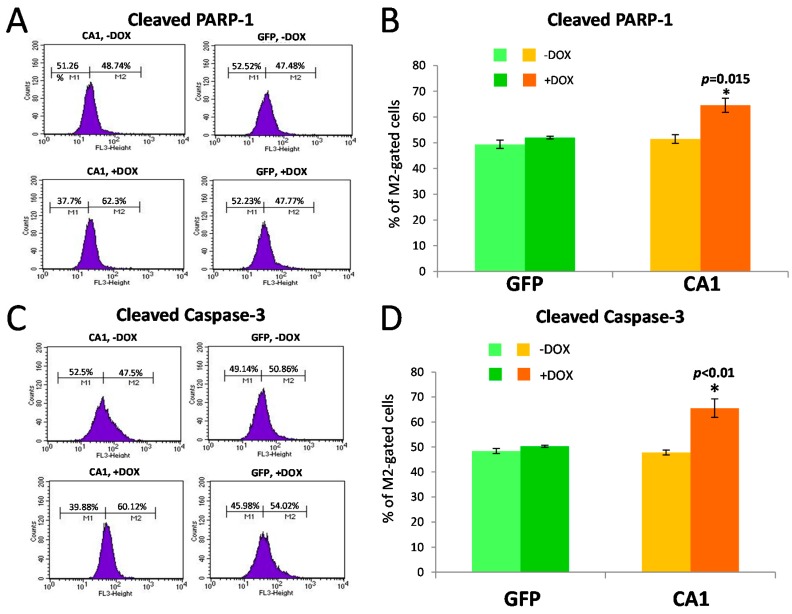
Expression of CA1 in HEK293 cells induces activation of apoptosis indicated by the cleavage of both PARP-1 and Caspase-3. CA1 expression was induced by 0.25 μg/mL doxycycline (DOX) for 96 h and HEK293 cells were analyzed via FACS using PARP-1 and Caspase-3 antibodies which recognized only cleaved protein fragments, in absence (−) or presence (+) of DOX. The expression of GFP was used as the control. (**A**,**C**) Representative FACS profiles of measuring cleaved PARP-1 (**A**) and cleaved Caspase-3 (**C**) in HEK293 cells; The histogram is plotted with the cleaved Caspase-3 or cleaved PARP-1 fluorescent intensity (*x*-axis) against cell counts (*y*-axis). M1 and M2 gates mark the cell population used to observe the fluorescence shift across the *x*-axis. The percentage of the cells in M1 and M2 was indicated directly above the gated line; (**B**,**D**) Bar graphs representing the degrees of PARP-1 (**B**) and Caspase-3 (**D**) cleavage as indicated by the percentage of M2 gated cells. The data are the average of three independent experiments and expressed as “Mean ± SEM”. * indicates *p* value < 0.05.

When these cells were analyzed by FACS for the detection of the cleaved PARP-1 and Caspase-3, two molecules involved in the activation of apoptosis, there were significant increases in the cleavage of both PARP-1 (Figure 6A,B) and Caspase-3 (Figure 6C,D) induced by CA1 at 96 h. The effect is specific as no changes in cleaved PAPR-1 and Caspase-3 were seen with overexpression of GFP at the same time (Figure 6). The reduced cell survival and activation of two apoptotic markers, PARP-1 and Caspase-3, upon CA1-induction demonstrate that CA1 can cause cellular toxicity.

## 3. Discussion

Our study has discovered that cytosolic CA1 is expressed in spinal cord motor neurons. Unexpectedly, the pattern of CA1 immunoreactivity and its co-localization with PDI in motor neurons indicates that a subpopulation of CA1 proteins is associated with the ER. More importantly, while the known CNS-expressing cytosolic CA2 is not changed in ALS, CA1 was significantly increased with more pronounced change in the membrane-associated fraction in ALS spinal cord (Figure 3). The pattern of CA1-immunostaining was also altered in ALS spinal cord motor neurons (Figure 4). To further understand the potential involvement of CA1 in ALS pathology, we provide evidence that CA1 can induce apoptosis in HEK293 cells.

CAs are essential enzymes in cells without which many physiological functions and metabolism cannot operate normally. It is therefore recognized that different isoforms of CAs are present in all cells to regulate both the intracellular and extracellular pH for the overall cellular pH homeostasis and functions. It also appears that more than one isoform of CAs may be present in any given cell to serve physiological functions. There was no detailed information on the identification of all CA isoforms expressed in neuronal cell-types.

CA1 is less well studied than CA2 due to the fact that it has lower catalytic activity [6]. CA1 expression has been observed in several peripheral tissues, while its expression in the CNS is reported to be extremely low in comparison with CA2 [48]. Our study is the first to demonstrate that CA1 expression is readily detectable in spinal cord motor neurons, adding yet new information to the diverse expression of CA isoforms, especially in the CNS. With the detection of its expression in motor neurons, the potential function of CA1 can be studied.

We detected increased levels of CA1 in ALS spinal cord, whereas CA2 exhibited no such changes (Figure 3). We also observed CA1 within punctate structures in the motor neurons (Figure 1) that co-localized with a marker for the ER (Figure 2). Thus, CA1 joins other cytosolic molecules whose associations with subcellular structures have also been reported. One of them is SOD1, which was first reported to be localized in the intermembrane space of mitochondria [57] and more recently to be palmitoylated [58]. This palmitoylation was associated with the immature SOD1 conformation, targeting it to membrane structures. The increased targeting to mitochondria was observed for mutant SOD1s that cause ALS, suggesting this non-cytosolic form of SOD1 may be directly involved in disease mechanisms [58].

The observation of CA1’s association with the ER bears a striking resemblance to the phenomenon of membrane-targeted SOD1 and its potential involvement in ALS. This conjecture is further supported by the fact that the level of membrane-associated CA1 sub-population was increased in ALS, and the subcellular distribution in surviving motor neurons exhibited a less pronounced punctate immunostaining (Figure 4). It is not resolved in our current study whether there is a direct link between CA1 and the ER stress pathway that is relevant for ALS; our data provide potential reasons to hypothesize that CA1 might be able to modulate ER stress responses or, alternatively, elevation of CA1 might be directly or indirectly associated with ALS-induced ER stress. Future studies will explore this potential role of CA1 in motor neurons. Our data also imply that CA1 serves different functions from CA2 in the human spinal cord, as their expression pattern did not overlap and we did not detect any changes in CA2 during ALS.

Our in vitro data demonstrate that CA1 can induce apoptotic pathways in HEK293 cells and they reveal a new function of CA1. This also is the first evidence to show that the intracellular CA1 can reduce cell survival, which would be via a different mechanism from the increased extracellular CA1 released during retinopathy [43] or up-regulated in other diseases [42,46]. This finding can go well (if proven) with the proposed ER signaling pathway which is known to cause activation of apoptosis in the form of PARP-1 and/or Caspase-3 activation.

In summary, we have determined that CA1 is expressed in spinal cord motor neurons and partially co-localizes to the ER in control motor neurons. CA1 protein levels are increased in ALS spinal cord and CA1 immunoreactivity was observed in a more diffuse cytoplasmic distribution in ALS motor neurons. Our data provide a foundation to further test the hypothesis of potentially linking CA1 to ER-associated activation of motor neuron death in ALS pathogenesis.

## 4. Experimental Section

### 4.1. Human Tissues

Human autopsy samples were from The Brain and Tissue Bank for Developmental Disorders of the National Institute of Child Health and Human Development (Baltimore, MA, USA) and Forbes-Norris MDA/ALS Research Center (San Francisco, CA, USA). Informed consents were obtained from all subjects before sample collections. Handlings of all human samples were in accordance with the approved protocol by California Pacific Medical Center Internal Board Review (IRB). (Protocol code 25.012; date of approval: 19 January 2005).

### 4.2. Antibodies and Other Reagents

The antibody to SOD1 was as described [55]. Antibodies against CA1 including GαCA1 (ab6619), mαCA1 (ab54912), RbαCA1 (ab108367), PDI (ab5484), mito (ab3298), and Giantin (ab24586) were from Abcam (Cambridge, MA, USA). HRP-GαCA1 (#200-1354) and the antibody against CA2 HRP-RbαCA2 (#200-403-136) was from Rockland (Gilbertsville, PA, USA). The actin antibody (A2066) was from Sigma (St. Louis, MO, USA). The monoclonal antibodies against neurofilaments SM31 and SM32 were from Sternberger-Meyer Immunochemical Inc. (Jarettsville, MD, USA). Both recombinant human CA1 (#2180-CA) and CA2 (#2184-CA) were purchased from R&D Systems (Minneapolis, MN, USA). The Broad Range Molecular Weight Standards (#161-0317) were from BioRad (Hercules, CA, USA). SYRPO-Ruby (#S12000) was from Thermo Fisher Scientific (Waltham, MA, USA). Polybrene (#H9268) was from Sigma (St. Louis, MO, USA).

### 4.3. Cell Cultures and Establishing Inducible Stable Cell Lines

HEK293 cells were cultured and maintained in Dulbecco’s Modified Eagle Medium (DMEM) containing 10% Fetal Bovine Serum (FBS), 0.1% Penicillin/Streptomycin at 37 °C/5% CO_2_. Stable cell lines with inducible expression of the proteins were established using a modified TRIPZ lentiviral inducible system consisting of pSPAX2 (lentivirus packaging vector), pMD2.G (lentivirus envelope vector) and pTRIPZ (protein expression vector) plasmids. All plasmids were kindly provided by Dr. Ferdinand Kappes. The cDNA of GFP or CA1 was cloned into the pTRIPZ plasmid.

To produce lentiviral particles, lipofectamine-mediated transfection with a mixture of 900 ng of pSPAX2, 100 ng of pMD2.G, and 1 μg of pTRIPZ in 6 μL of lipofectamine reagent (#11668019, Life Technologies, Carlsbad, CA, USA) were added to 50%–70% confluent HEK293 cells in a 6 cm dish. At 18 h post-transfection, the cell medium was replaced with the medium containing 30% fetal bovine serum (FBS) and 1% Penicillin/Streptomycin. The medium which contained lentiviral particles were harvested at 40 h post-transfection and filtered through 0.45 μm filters for obtaining the viral supernatant. Fresh medium containing 30% FBS was added back to the cells.

To transduce cells, HEK293 cells were seeded 24 h prior to the viral transduction in 10 cm dishes. When they reached 10%–30% confluency, the cells were transduced with the viral supernatant containing 8 μg/mL polybrene for 6 h after which the medium was changed to normal cell growth medium and incubated at 37 °C/5% CO_2_. At 12 h, the viral medium was harvested and filtered again. The transduction procedure was then carried out for the second round. The cells were transduced with the viral supernatant for 6 h again and the viral medium was replaced with normal cell growth medium after transduction. After 24 h, the transduced cells were selected in the presence of 1 μg/mL puromycin. After 2 weeks, pure stable cell lines were obtained from transduced cells surviving from the puromycin selection. After the stable cell lines have been obtained, the cells were maintained in the growth medium containing 0.25 μg/mL of puromycin.

### 4.4. Immunohistochemistry and Immunofluorescent Labeling 

The spinal cords from autopsy samples were dissected out and fixed in 4% formaldehyde. Tissues were further processed, embedded in paraffin, and sectioned at 7 μm thickness. Paraffin sections were processed for either immunohistochemistry using the DAB (3,3′-diaminobenzidine tetrahydrochloride, Sigma, St. Louis, MO, USA) method with the Vector ABC Kit (Vector Laboratories, Burlingame, CA, USA) or the immunofluorescent labeling. All sections were deparaffinized, incubated in 20% H_2_O_2_/10% methanol solution to quench any endogenous peroxidase activity (for the DAB method only), heat-boiled by microwaving to expose the epitopes, and blocked in 5% normal serum in PBS/0.01% Triton for 1.0 h at room temperature. Subsequently, sections were incubated with primary antibodies in PBS/1% normal serum overnight at 4 °C. For the DAB-labeling, the sections were continued for incubation with biotinylated secondary antibodies and substrates A and B. Immunoreactive signals were visualized by adding DAB for a brown color-development followed by counter-staining with hematoxylin. For the immunofluorescent labeling, the sections were continued for incubation with Cy3 or Cy5-conjugated secondary antibodies (Jackson ImmunoResearch Laboratories, West Grove, PA, USA). All images were captured on the upright Nikon microscope (ECLIPSE Ni-E, Nikon Inc., Melville, NY, USA) equipped with a CCD (charge coupled device) camera.

### 4.5. Western Blot Analysis 

Spinal cord tissues were fractionated as described [55]. The protein concentration was determined using the BCA kit (#23225, Thermo Fisher Scientific, Waltham, MA, USA). Cytosolic and microsomal proteins were separated on SDS-PAGE gels, transferred to nitrocellulose membranes, and incubated with the primary antibodies using the ECL (enhanced chemiluminescence) detection method (GE Healthcare, Piscataway, NJ, USA). The intensities of the immunoreactive signals were quantified using the ImageJ (an open source software developed by National Institute of Health, Baltimore, MD, USA).

HEK293 cells in 12-well plates were lysed using the sample loading buffer (61.3 mM Tris pH 8.0, 2% SDS, 10% glycerol, 0.0025% bromophenol blue, 2.5% β-mercaptoethanol) and centrifuged for 30 min at 4 °C. Equal volumes of protein samples were resolved on 12% SDS/PAGE. Proteins were transferred to nitrocellulose membranes, blocked in 5% milk solution in TBST, incubated with the primary antibodies followed by 608-conjugated anti-rabbit secondary antibody (#9263223, Li-Cor Bioscience, Lincoln, NE, USA). The signals were visualized on Odyssey Infra-red Imager (Li-Cor Biosciences, Lincoln, NE, USA). 

### 4.6. Cell Survival Assay

Cells were plated in a 96-well plate. The WST8 (Dojindo Laboratories, Rockville, MD, USA) assay was carried out to measure cell viability. A total of 10 μL of the WST8 reagent was added to each well with 100 μL of the medium. The absorbance at 450 nm was taken after 2 h incubation at 37 °C/5% CO_2_ on a microplate reader (BioTek, Winooski, VT, USA).

### 4.7. Flow Cytometry Analysis

Flow cytometry analysis was used to determine the extent of apoptotic activation induced by CA1 in HEK293 at 96 h post-induction of protein expression. Cells were collected by centrifugation at 500× *g* for 5 min and fixed by re-suspension with PBS solution containing 4% Paraformaldehyde at room temperature for 15 min. Cells were then pelleted, re-suspended in 90% methanol and 10% PBS solution, and incubated at −20 °C for 30 min to permeabilize membranes. Cells were washed twice with freshly made PBS/1% Bovine Serum Albumin (BSA) and blocked in the same solution for 20 min at room temperature. After blocking, cells were washed with 1% BSA/PBS and incubated with anti-Caspase-3-Alexa Fluor 647 (BD, #560626, San Diego, CA, USA) or anti-PARP-1-Alexa Fluor 647 antibodies (BD, #558710, San Diego, CA, USA) to detect apoptotic changes. The dilutions for both anti-PARP-1 and anti-Caspase-3 antibodies used in the experiment were 1:200 as recommended by the manufacturer. Fluorescence signals were collected in the log mode using a FACSCalibur (BD, San Diego, CA, USA) and data acquisition was done using the CellQuest Pro software (BD, San Diego, CA, USA). Analysis of cell populations were performed on events gated according to the forward light scatter/side scatter parameters.

### 4.8. Statistical Analysis

The statistical significance between the two data sets was assessed using the Graph-Pad Prism software, version 5.04 (GraphPad, San Diego, CA, USA). Two-tailed *t*-tests were used and the statistical significance was set at *p* < 0.05.

## Supplementary Materials

Supplementary materials can be found at www.mdpi.com/1422-0067/17/11/1820/s1.
