# Supplementary material for: Expression of Carbonic Anhydrase I in Motor Neurons and Alterations in ALS
Source: Int J Mol Sci. 2016 Nov 1;17(11):1820. doi: 10.3390/ijms17111820 (PMC5133821; doi:10.3390/ijms17111820)
Supplement: Supplementary file 1 [file ijms-17-01820-s001.pdf]

# Supplementary Materials: Expression of Carbonic Anhydrase I in Motor Neurons and Alterations in ALS

**Xiaochen Liu, Deyi Lu, Robert Bowser and Jian Liu**

**Table S1.** Samples used for immunohistochemical staining of paraffin sections in Figures 2 and 4.

| Label | Sex | Age (Year/Day) | Race | Post Mortem Intervals (h) | Disorder | Cause of Death |
|---|---|---|---|---|---|---|
| A | Male | 55/32 | Caucasian | 24 | Control | Arteriosclerotic Cardiovascular Disease |
| B | Male | 27/42 | Caucasian | 15 | Control | Accident, multiple injuries |
| C | Male | 26/253 | Caucasian | 18 | Control | multiple injuries |
| D | Male | 57 | Caucasian | N/A | ALS | complication of disorder |
| E | Female | 87/176 | Caucasian | 18 | ALS | complication of disorder |

N/A: not known.

**Table S2.** Samples used for the Western analysis in Figure 3.

| Label | Sex | Age (Year/Day) | Race | Post Mortem Intervals (h) | Disorder | Cause of Death |
|---|---|---|---|---|---|---|
| Control_1 | Male | 15 | African American | 13 | Control | Accident, multiple injuries |
| Control_2 | Male | 14/308 | Caucasian | 16 | Control | Accident, multiple injuries |
| Control_3 | Male | 27/42 | Caucasian | 15 | Control | Accident, multiple injuries |
| Control_4 | Male | 55/32 | Caucasian | 24 | Control | Arteriosclerotic Cardiovascular Disease |
| Control_5 | Male | 26/253 | Caucasian | 18 | Control | Multiple injuries |
| SALS_1 | Female | 73/231 | Caucasian | 20 | ALS | complication of disorder |
| SALS_2 | Male | 69/53 | Caucasian | 22 | ALS | complication of disorder |
| SALS_3 | Female | 46/263 | Caucasian | 3 | ALS | complication of disorder |
| SALS_4 | Female | 57/70 | Caucasian | 14 | ALS | complication of disorder |
| SALS_5 | Male | 45/344 | Caucasian | 15 | ALS | complication of disorder |
| SALS_6 | Female | 56/17 | Caucasian | 6 | ALS | complication of disorder |
| SALS_7 | Female | 87/176 | Caucasian | 18 | ALS | complication of disorder |
| SALS_8 | Male | 49/39 | Caucasian | 9 | ALS | complication of disorder |

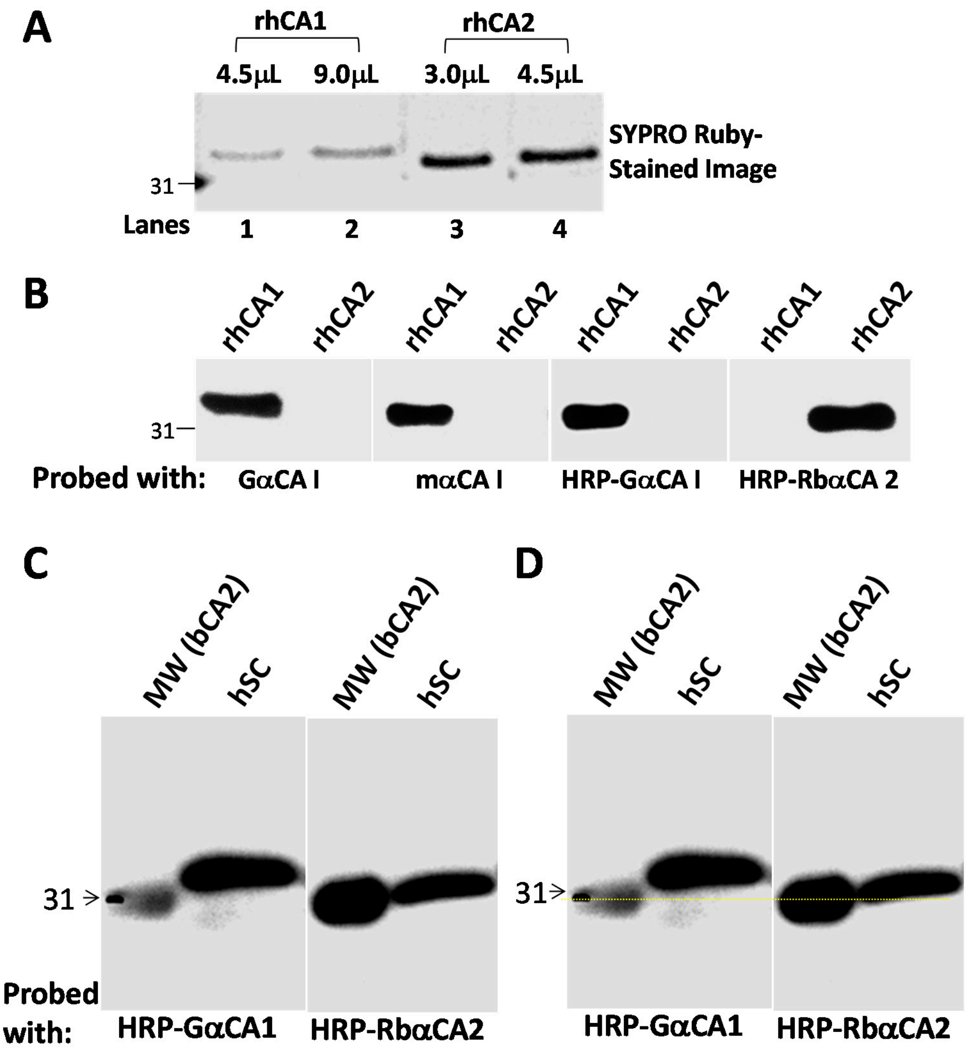


**Figure S1.** CA1 antibodies recognize human CA1 but not human CA2. (**A**,**B**) Commercially available recombinant His-tagged human CA1 and CA2 proteins were used for SYPRO-Ruby staining and Western analysis; (**A**) Two different amounts of CA1 (4.5 µL and 9.0 µL) and CA2 (3.0 µL and 4.5 µL) from the prepared protein samples were used and visualized by SYPRO Ruby-staining on SDS-PAGE; (**B**) Four identical Western strips with 9.5 µL of CA1 and CA2 proteins for each lane on each blot were probed with 3 different sources of CA1 antibodies (G$\alpha$CA1, m$\alpha$CA1, and HRP-G$\alpha$CA1) and one CA2 antibody (HRP-Rb$\alpha$CA2); (**C**) Identical blots with proteins from the human spinal cord (20 µg/lane) together with the MW which contains the bovine CA2 (bCA2) were analyzed by Western blot using HRP-G$\alpha$CA1and HRP-Rb$\alpha$CA2, respectively. The bCA2 shares 58% and 80.4% in amino acid sequence identity with human CA1 and CA2, respectively. The CA1 antibody recognized one hCA1 band and cross-reacted to a small degree with bCA2. The CA2 antibody recognized one hCA2 band and readily recognized bCA2; (**D**) The exact same data as (**C**) with a yellow-dotted line drawn across the center of bCA2 signal to illustrate the different positions of the CA1 and CA2 signals recognized by their respective antibodies using bCA2 as the reference. MW: Molecular Weight marker; hsc: human spinal cord.

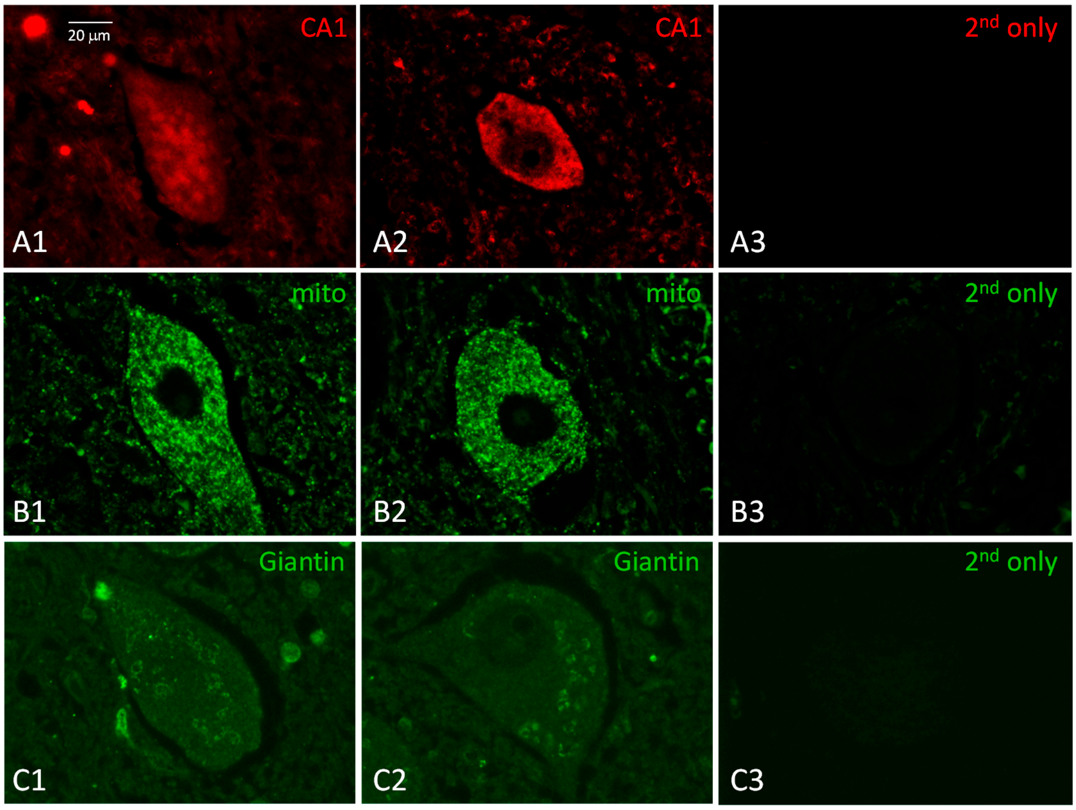


**Figure S2.** The pattern of CA1 immunoreactivity in human spinal cord motor neuron did not resemble those from mitochondria or Golgi. The control human spinal cord sections were immunofluorescently stained with antibodies against CA1 (GαCA1, **red**, **A1**,**A2**); and the molecular markers of subcellular organelles including mitochondria (**green**, mito, **B1**,**B2**); and Golgi (**green**, Giantin, **C1**,**C2**). The background staining images with the secondary antibodies only for each primary antibody were included on the most **right** column (2nd antibody only, **A3**, **B3**, and **C3**). The **white** scale bar indicates 20 μm.

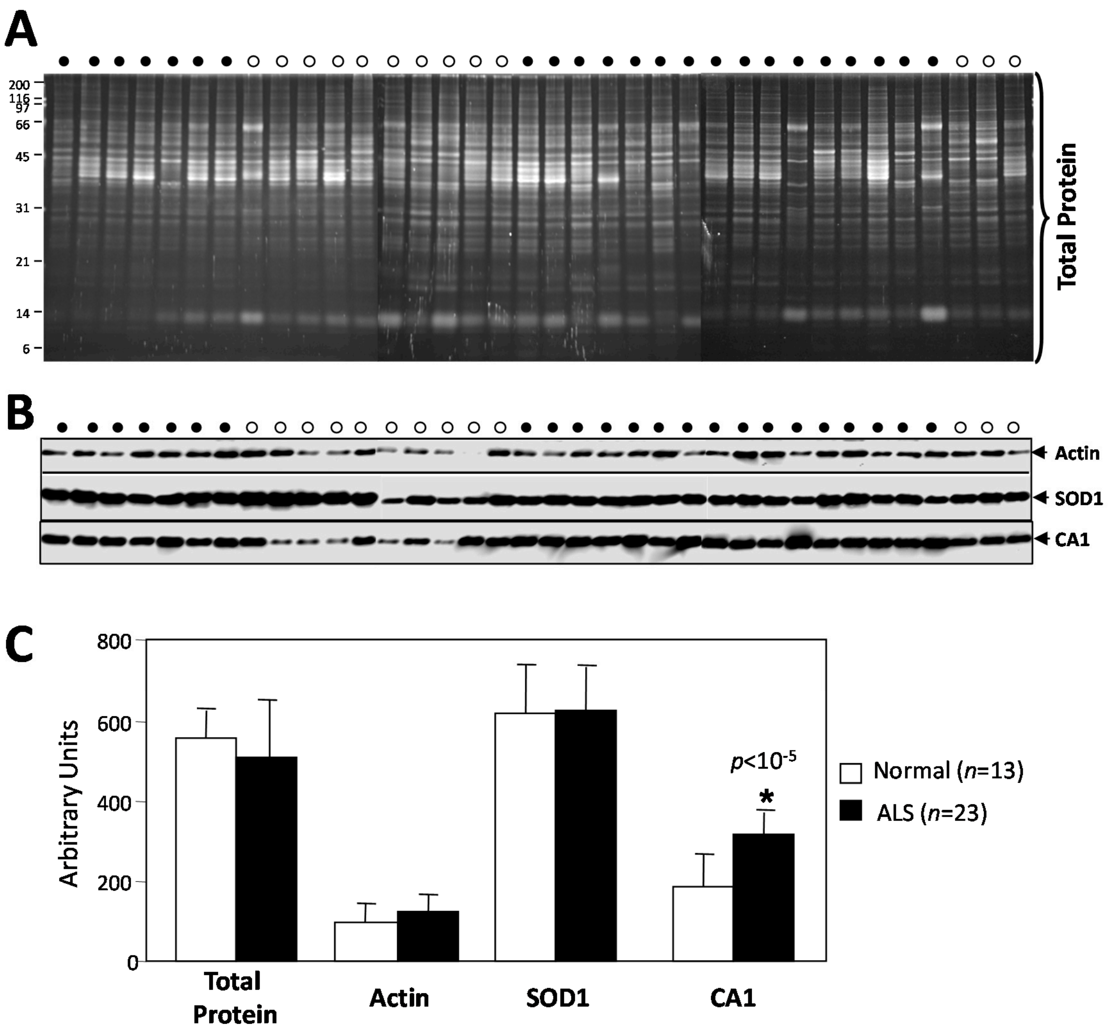


**Figure S3.** The levels of cytosolic CA1 proteins were increased in ALS spinal cords and cytosolic SOD1 protein levels behave similar to those of actin which can serve as the internal control for sample loading. Cytosolic proteins from both control (open circles) and ALS (filled circles) spinal cords were extracted as described and used for SYPRO-Ruby staining and the Western analysis. (**A**) An equal amount of 1.0 μg of proteins was loaded for each lane on 3 SDS-PAGE gels and stained with SYPRO-Ruby for visualization of the total proteins with the Broad Range Molecular Standards indicated on the most left; (**B**) An equal amount of 20 μg of proteins was loaded for each lane on 3 SDS-PAGE gels and processed for Western analysis with antibodies against actin and SOD1; (**C**) Quantitation of the intensities of the total protein in each lane (**A**) and immunoreactive signals of actin, SOD1 and CA1 (**B**). The intensity of the total protein for each sample was normalized to the same amount Broad Range Molecular Weight Standards on each gel (not shown) so that the intensities of all samples can be combined and compared. A common spinal cord protein sample (not shown) was used for each Western blot and the intensity of the immunoreactive band for actin, SOD1, and CA1 was normalized to the corresponding actin, SOD1, and CA1 signal in the common sample so that the intensities of all samples can be combined and compared. The data are expressed as "Mean ± SD". There were no significant differences in the total amount of proteins loaded in each lane, nor in the immunoreactive intensities of either actin or SOD1, between control and ALS groups. Therefore, both actin and SOD1 can serve as the internal control for sample loading.
